# Metabolomic analysis reveals reliance on secondary plant metabolites to facilitate carnivory in the Cape sundew, *Drosera capensis*

**DOI:** 10.1093/aob/mcab065

**Published:** 2021-06-02

**Authors:** Christopher R Hatcher, Ulf Sommer, Liam M Heaney, Jonathan Millett

**Affiliations:** 1 Loughborough University, Loughborough, UK; 2 Agri-Tech Centre, Pershore College, Part of WCG, Pershore, UK; 3 Biocrates Life Sciences AG, Innsbruck, Austria

**Keywords:** *Drosera capensis*, secondary plant metabolites, carnivorous plant, plant–insect interactions, metabolomics

## Abstract

**Background and Aims:**

Secondary metabolites are integral to multiple key plant processes (growth regulation, pollinator attraction and interactions with conspecifics, competitors and symbionts) yet their role in plant adaptation remains an underexplored area of research. Carnivorous plants use secondary metabolites to acquire nutrients from prey, but the extent of the role of secondary metabolites in plant carnivory is not known. We aimed to determine the extent of the role of secondary metabolites in facilitating carnivory of the Cape sundew, *Drosera capensis.*

**Methods:**

We conducted metabolomic analysis of 72 plants in a time-series experiment before and after simulated prey capture. We used ultra-high-performance liquid chromatography–tandem mass spectrometry (UHPLC–MS/MS) and the retention time index to identify compounds in the leaf trap tissue that changed up to 72 h following simulated prey capture. We identified associated metabolic pathways, and cross-compared these compounds with metabolites previously known to be involved in carnivorous plants across taxa.

**Key Results:**

For the first time in a carnivorous plant, we have profiled the whole-leaf metabolome response to prey capture. Reliance on secondary plant metabolites was higher than previously thought – 2383 out of 3257 compounds in fed leaves had statistically significant concentration changes in comparison with unfed controls. Of these, ~34 compounds are also associated with carnivory in other species; 11 are unique to Nepenthales. At least 20 compounds had 10-fold changes in concentration, 12 of which had 30-fold changes and are typically associated with defence or attraction in non-carnivorous plants.

**Conclusions:**

Secondary plant metabolites are utilized in plant carnivory to an extent greater than previously thought – we found a whole-metabolome response to prey capture. Plant carnivory, at the metabolic level, likely evolved from at least two distinct functions: attraction and defence. Findings of this study support the hypothesis that secondary metabolites play an important role in plant diversification and adaptation to new environments.

## INTRODUCTION

Secondary plant metabolites are integral for plant fitness and are a key aspect of plant evolutionary adaptation (Duplais *et al.*, 2020). They are involved in a wide range of key plant processes such as growth regulation (Erb and Kliebenstein, 2020), pollinator attraction (Hartmann, 2007; Kessler and Baldwin, 2007), facilitating interactions between conspecifics (Catola *et al.*, 2018), competitors and symbionts (van Dam and Bouwmeester, 2016), and as defence against abiotic and biotic stress (Agrawal, 2011). Their importance is clearly defined for only a narrow selection of these processes, predominantly pollination, stress and defence (Kessler and Baldwin, 2007; Ramakrishna and Ravishankar, 2011; Jander, 2014); the extent of their function outside of these processes is, however, not well understood. Plant carnivory is an example of a life-history strategy that relies on secondary metabolites (Hatcher *et al.*, 2020), and for which convergent evolution of secondary metabolites appears to be likely. The extent to which carnivorous plants rely on secondary metabolites and the diversity of compounds used to facilitate carnivory remain an important area to be determined.

Carnivorous plants are a diverse, polyphyletic group of flowering plants adapted, in general, to nutrient-poor environments (Darwin, 1875; Albert *et al.*, 1992; Ellison and Gotelli, 2001). Plant carnivory involves adaptation to facilitate the key processes of prey attraction, capture, digestion and assimilation (Ellison and Adamec, 2018). The morphological and physiological basis for these adaptations is well known (Gibson and Waller, 2009; Król *et al.*, 2012; Gaume *et al.*, 2016), with some clear examples of convergent and divergent evolution (e.g. pitcher plants, Thorogood *et al.*, 2017). There is clear evidence that secondary metabolites play a key role in some of these processes, such as olfactory cues, prey capture signalling, trap movement and defence of plant tissue from decaying prey (see review by Hatcher *et al.*, 2020). An understanding of biochemical profiles of the process of plant carnivory is, however, currently not determined. Most studies focusing on the role of secondary metabolites in plant carnivory are targeted analyses of known compounds. Jasmonates, for example, are typically involved in stress response but in *Drosera* spp. they instigate leaf bending within an hour following prey capture (Krausko *et al.*, 2017). We lack, however, a more focused understanding of the biochemical responses to prey capture of a carnivorous plant. Unsupervised metabolomic methods provide an effective strategy for determining the extent of involvement of secondary metabolites in carnivorous plants. Understanding the scale of the role of metabolites in a plant’s carnivory provides insight into the importance of secondary plant metabolites for diversification into new environments and the capacity for these compounds to act as conduits for evolutionary adaptation in areas outside of pollination, stress and defence.

To establish the potential extent of the biochemical role of secondary plant metabolites in carnivory, we measured the metabolic profile of the carnivorous herb Cape sundew, *Drosera capensis*, before and after the addition of rehydrated *Drosophila melanogaster* powder (‘insect substrate’ herein) to the traps. Up- or downregulation of compounds following insect substrate addition indicates the involvement of that compound in carnivory (as per Kreuzwieser *et al.*, 2014). We predicted that there is a whole-leaf metabolic response to substrate addition incorporating multiple secondary metabolites to facilitate carnivory. Specifically, we hypothesize that (1) compounds involved in leaf bending, e.g. jasmonates, will increase in concentration within 45 min and the increase will be sustained for the remainder of the experiment; (2) compounds involved in attraction will decrease in concentration following nutrient addition as the benefit of these compounds is reduced; and (3) there will be a latency of certain compounds before upregulation, these compounds being suggested to be compounds involved in the digestion or assimilation of nutrients from prey.

## MATERIALS AND METHODS

### Plant rearing and preparation

*Drosera capensis* L. plants were purchased from an established nursery. Prior to this, seeds were grown in an open polytunnel on a standard medium of peat:sand:perlite (6:1:1 respectively) in individual pots. Plants were able to catch available prey during growth to maturity. On 1 July, 4 weeks prior to the experiment, 500 mature individuals were translocated to a greenhouse at Loughborough University. Exposure to prey and prey items on leaves were restricted as much as possible; the few prey that were captured were removed during daily inspections. The greenhouse was maintained at ambient temperature, with supplementary heating to prevent the temperature dropping below 18 °C, additional fluorescent lighting on a light:dark cycle of 12:12 h was used and plants were watered regularly with de-ionized water when required. To prevent allocation to carnivory being confounded by reproductive investment, flower stems were removed when visible during this time up to the final week before the experiment, when removal of stems may have mitigated a stress response. Plants were selected at random from the 500 individuals in the greenhouse for use in this experiment.

### Experimental design

The metabolic response of *D. capensis* to prey capture was determined by adding insect substrate to one leaf-trap of an individual plant; this leaf was subsequently harvested for analysis. Leaf response to prey is known to initiate within the first 6 h after prey capture but can take place over multiple days or weeks to completion (Adamec, 2002; Nakamura *et al.*, 2013; Krausko *et al.*, 2017). To capture these short- and longer-term plant responses, we measured changes across a time-series of harvests from insect substrate addition. Plants were harvested at 0.75, 1.5, 3, 6, 24, 48 and 72 h after insect substrate addition. Circadian variability in the metabolome of plants is common (Kim *et al.*, 2017). We therefore staggered insect substrate addition throughout the day (prey additions from 0515 to 1250 h) to keep harvest times in the shortest possible time window. Plant harvests were therefore all carried out between 1030 and 1330 h. Each time interval had six replicates. These replicates were spread evenly throughout the harvest period and at least one unfed control was harvested within 10 min of a fed plant being harvested to account for natural circadian changes and comparison of the metabolite profile with that of unfed plants (total number of controls = 32). There was a total of 74 plant samples. Due to the amount of work involved, the experiment was carried out in two overlapping sections. The first started on day 1 (2 August) and finished on day 4, and the second started on day 2 and finished on day 5 (6 August).

### Prey addition

To simulate an accurate and standardized response of *D. capensis* to prey capture, fruit fly powder (*Drosophila melanogaster*) was used as the prey addition (fed) treatment using the protocol outlined by Gao *et al.* (2015). Fruit flies were reared on a standard medium, freeze-dried and ground into a homogenized powder using a ball mill. Insect substrate addition consisted of 25 mg of fruit fly powder in 100 µL of de-ionized water spread evenly and carefully across the trap using a pipette, intentionally stimulating the leaf-trap tentacles mechanically.

### Plant harvests

At each harvest, leaf and root tissue was cut from the plant and washed immediately in de-ionized water to remove prey residue and soil. The leaf was then flash-frozen in liquid nitrogen (−196 °C). The time from cutting to freezing was always <30 s. Tissue samples were then weighed before being placed in a Precellys homogenizer tube (Bertin Technologies, Sain Quentin en Yvelines, France) in a −80 °C freezer until analysis.

### Sample preparation, UHPLC-MS analysis and processing for metabolite profile

For a more in-depth description of these methods, please see Supplementary Data Methods Detail and data available at Hatcher *et al.* (2021). Monophasic extractions used acetonitrile:methanol:water 2:2:1 as the final solvent. Equal volumes of the supernatant were stored at −80 °C. LC–MS samples were reconstituted in 50 µL of 20 % aqueous methanol each, and 10 μL each was pooled into one quality control (QC) sample per sample type, giving 16 QC samples in total.

The samples were run in controlled randomized order, with QC samples equidistant between them. They were analysed by ultra-high-performance liquid chromatography-mass spectrometry (UHPLC-MS). Quality control sample 02 contains data-dependent MS/MS data and is used with the retention time index to annotate metabolites. Data were collected in positive ion and profile mode, *m*/*z* 100–1000, at 70 k resolution (see Supplementary Data Fig. S1 for total ion chromatogram for a blank and a QC run).

Data processing and QC methods followed guidelines outlined by Di Guida *et al.* (2016) and Kirwan *et al.* (2014) using NBAF-B in-house scripts in MatLab (v8.1; The MathWorks, Natick, MA, USA), the SIMStitch pipeline. Briefly, an R (3.2.0)-based XCMS/CAMERA script was used for alignment and resulted in an intensity matrix in a csv file (9309 features). Following replicate and blank filtering, samples were filtered with a 2-ppm mass error and a 75 % filter. Data were normalized using probabilistic quotient normalization (PQN) and missing values were filled using the *k*-nearest neighbours (KNN) algorithm (*k* = 5). This matrix was used for univariate statistics including fold changes. Generalized logarithm (g-log) transformation was applied to all samples. This matrix was used for multivariate statistics.

### Statistical analysis and identification of metabolites

We initially used principal component analysis (PCA) to assess the overall metabolic differences among the control and time-after-feeding treatments in an unbiased manner using the ‘prcomp’ function in R (R Core Team, 2016). Following this, there was obvious separation of the treatments into three groups: controls, ≤6 h and ≥24 h. Further analyses were, therefore, conducted for these three pooled groups. PCA of the samples highlighted a clear single anomalous control sample, which we excluded from all analyses.

We subsequently performed three separate supervised multivariate analyses using orthogonal partial least-squares discriminant analysis (OPLS-DA) using Simca-P+ (v14, Umetrics Umeå, Sweden) to find the direction of maximum covariance: between controls and both treatments (≤6 h or ≥24 h); among grouped treatments (≤6 h and ≥24 h); and between all three groups of treatments. OPLS-DA separates the difference between groups of observations by rotating PCA components such that maximum separation among groups is obtained and identifies variables that are most contributing to this separation. Simca-P+ validates the OPLS-DA model by removing one-seventh of the data and producing a model for the six-sevenths of remaining data. The new data are then predicted using the new model, continuing in this process until all the data have been predicted. We evaluated the contribution of each metabolite to the separation of treatments using S-plots and derived metabolites of biological relevance indicated from these plots. S-plots were analysed for metabolites that had the greatest selectivity and sensitivity in discriminating between the data, presented as points at the upper right and lower left corners of the plots (Supplementary Data Figs S2–S9).

We used univariate statistical analyses to identify changes in individual mass-spectral signals between unfed (controls) and grouped fed treatments (≤6 h’ or ≥24 h). A series of filters (ANOVA, *t*-tests and fold changes) were applied to the data. Metabolites that passed all three filters (i.e. were statistically significant in ANOVA, *t*-test and with a fold change greater than ±2) are presented and considered in more detail.

Firstly, we used analysis of variance (ANOVA) to determine if any compounds across all fed treatments changed significantly from the unfed controls. A Benjamini–Hochberg correction was applied to limit the false discovery rate (FDR) to 5 %. These identified compounds were compared with previously identified metabolites in other studies highlighted in Hatcher *et al.* (2020). Following this, we used multiple Welch’s *t*-tests to determine pairwise differences between controls and the ≤6 h or ≥24 h treatment group. We used Benjamini–Hochberg correction to control the FDR of 5 % to correct for multiple hypothesis testing of all treatment peak intensities compared with control to identify any changes in compound intensities across all treatments due to the large number of tests. These were then combined with log-fold changes to identify the significant and intense signal changes between control samples and the ≤6 h or ≥24 h treatments presented as volcano plots. Conservative thresholds of ±2 for the log_2_ fold change and 2 for the −log FDR-adjusted *P* value (*P* < 0.01) were used to highlight those features that showed the largest differences (Grace and Hudson, 2016). Of the significant compounds (high statistical significance and fold change), the 100 most significant compounds are presented in a heat map to illustrate plant metabolome response.

Metabolites that are highlighted by OPLS-DA as well as univariate analyses as statistically significant (as defined above) were cross-examined (Supplementary Data Table S1) and presented in box plots to inspect individual metabolite patterns.

### Annotation summary and pathway analysis

Annotation of metabolites were assigned from tandem MS (MS/MS) and metabolite databases. We used the in-house MIPack software to match 419 signals of the total of 3257 signals to the BioCyc/*Arabidopsis thaliana* database (5 ppm error), and 1290 signals up to *m*/*z* 600 to the KEGG database with 2 ppm error and including molecular formula search (as per Weber and Viant, 2010). Choline was annotated manually.

Compounds annotated and found to be biologically important from the analyses were inserted into the MetaboAnalyst pathway generator. MetaboAnalyst was implemented using the PrimeFaces library (v6.1) based on the JavaServer Faces Technology. The communication between Java and R is established through TCP/IP using the Rserve program (Li *et al.*, 2018). The pathway library selected was ‘*Arabidopsis thaliana*’. The over-representation analysis method was ‘hypergeometric test’. The node importance measure for topological analysis was ‘relative betweenness centrality’.

### Metabolite comparisons with other carnivorous plants

We cross-checked the compounds with a library of metabolites that have previously been associated with a role in carnivory (Hatcher *et al.*, 2020). Exact compounds or isomers were identified and their regulation before and after prey capture and the statistical significance of these changes in the experiment were produced in a table and compared with previously annotated compounds. Metabolites derived from carnivorous plants purely for pharmaceutical use are not discussed in this paper as their derivation is stimulated under conditions not analogous to the plant’s ecology and therefore do not assist in the identification of compounds likely to function in plant carnivory.

## RESULTS

In response to simulated prey capture, *D. capensis* upregulated and downregulated a large number of secondary plant metabolites over the course of 72 h. Of the 3257 analytical features present, statistically significant (ANOVA, adjusted *P* < 0.05) changes in intensity were found in 2383 peaks in at least one time point compared with controls (Supplementary Data Table S2). Among the 2383 compounds where change was detected, some clear patterns were identified. Generally, compounds that were upregulated increased in concentration within 0.75 h following substrate addition. After this point, in general, concentrations stopped increasing and the elevated concentration was maintained for the duration of the experiment. In some cases, metabolites rapidly increased at 0.75 h, then slowly increased for the duration of the experiment; other metabolites had a more consistent rate of increase over time which lasted the duration of the experiment. For a small proportion of compounds, increases in concentration were very large; >20 compounds had 10-fold increases and were statistically significant compared with controls, and 12 of these had >30-fold increases compared with controls. Decreases in concentration of downregulated compounds were mostly small in the first 6 h, followed by much larger decreases in concentration for the remaining 66 h. In very few cases, there were compounds that decreased in concentration initially, and then had an overall increase in concentration by the end of the experiment.

### Multivariate analyses

**Fig. 1.**
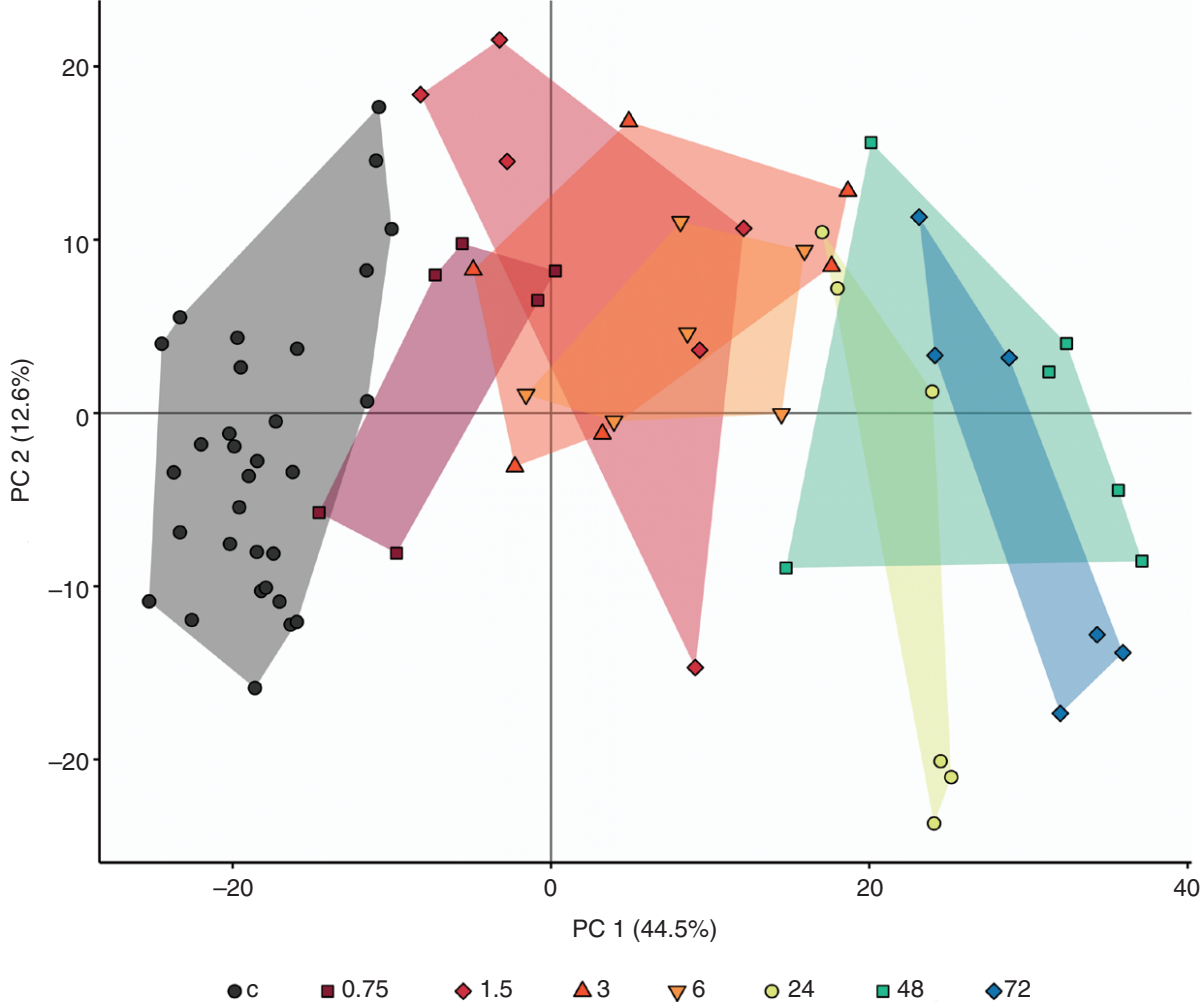
Ordination of the first two principle components from a PCA of metabolite profiles of *D. capensis* following insect substrate addition, with unfed controls. Treatments indicated are hours after substrate addition (c, unfed controls). Presented are the PC1 and PC2 scores for each plant, with convex hull colour for each treatment group. Different symbols represent each treatment time point. The presented PCA is without inclusion of the anomalous result (one control sample).

The PCA of the metabolome of plant samples showed a clear separation between treatment time points along PC1, increasing in time from left to right. PCA axes 1 and 2 explained 57·1 % (44·4 and 12·7 %, respectively) of the variance. Control groups were clustered at the left side, shorter times (0, 75, 1.5, 3, 6 h) clustered in the middle of the ordination, and longer treatment times (24, 48, 72 h) clustered at the right side of the ordination (Fig. 1). Further analysis, therefore, focused on compounds within these three clustered time range groups.

S-plots of metabolites between controls and ≤6 h highlighted 28 upregulated and 8 downregulated compounds. S-plots of metabolites between controls and ≥24 h highlighted ten upregulated and four downregulated compounds. OPLS-DA comparing ≤6 h with ≥24 h highlighted 13 upregulated and three downregulated compounds in ≥24 h compared with ≤6 h . A final OPLS-DA comparing all three groups (unfed, ≤6 h after feeding and ≥24 h after feeding) highlighted four compounds that were highly associated specifically with the ≥24 h after feeding group (Supplementary Data Figs S2–S9; annotated metabolites from these analyses are compiled in Supplementary Data Table S1).

### Compounds with high fold changes and importance (volcano plot)

**Fig. 2.**
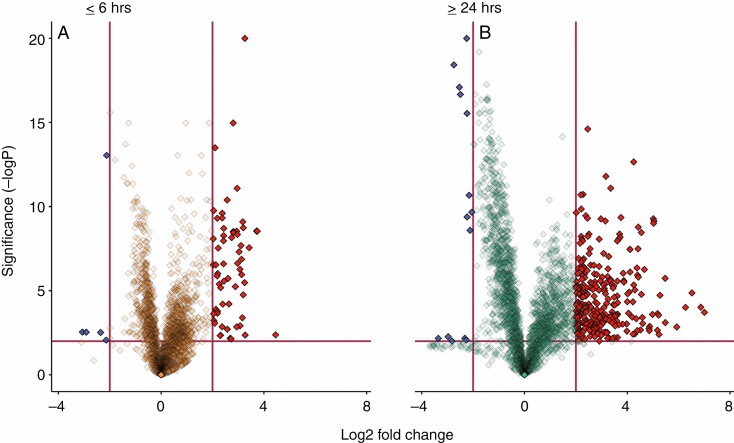
Number and proportion of discriminatory features in treatment groups compared with the control group. Shown here are log fold changes (*x*-axis), in comparison with unfed control plants, and the *y*-axis displays the negative log of the *P*-value from a two-sample *t*-test. Data points that are far from the origin (near the top or far left and right) are considered important variables with potentially high biological relevance. Blue and red features (downregulated and upregulated respectively) are those chosen based on the presented criteria of thresholds of 2 and −2 for the log_2_ fold change and 2 for the −log FDR-adjusted *P*-value (*P* < 0.01, indicated with red margins) to highlight those features that showed the largest differences (Grace and Hudson, 2016).

In the ≤6 h group compared with unfed controls, 62 compounds were upregulated and 5 downregulated, with a statistically significant fold change >4. In the ≥24 h group compared with unfed controls, 287 compounds were upregulated and 14 were downregulated, with a statistically significant fold change >4. All of the 62 upregulated compounds in the ≤6 h group were also identified among the 287 upregulated compounds in the ≥24 h group. None of the six downregulated compounds in the <6 h’ group was present in the statistically significant downregulated compounds in the ≥24 h group (Fig. 2 and Supplementary Data Table S1).

**Fig. 3.**
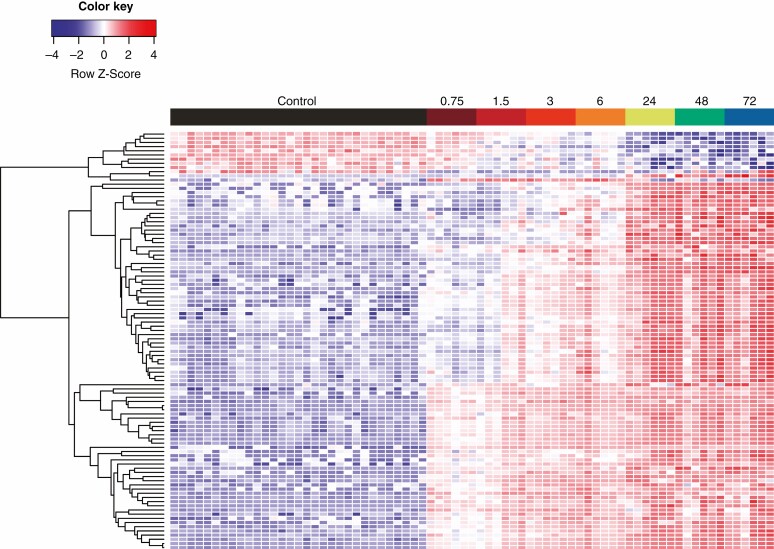
Metabolic patterns over time after prey substrate addition. Presented are the 100 most important features, based on statistical significance and fold change. Colour and colour intensity represent the *Z* score for the change in peak intensity from the mean of the compound (one compound per row). Each column represents a single plant; these are grouped according to treatment and harvest time point (randomly assigned within these). Each row represents a single metabolite grouped as a dendrogram by similarity in change of intensity.

**Fig. 4.**
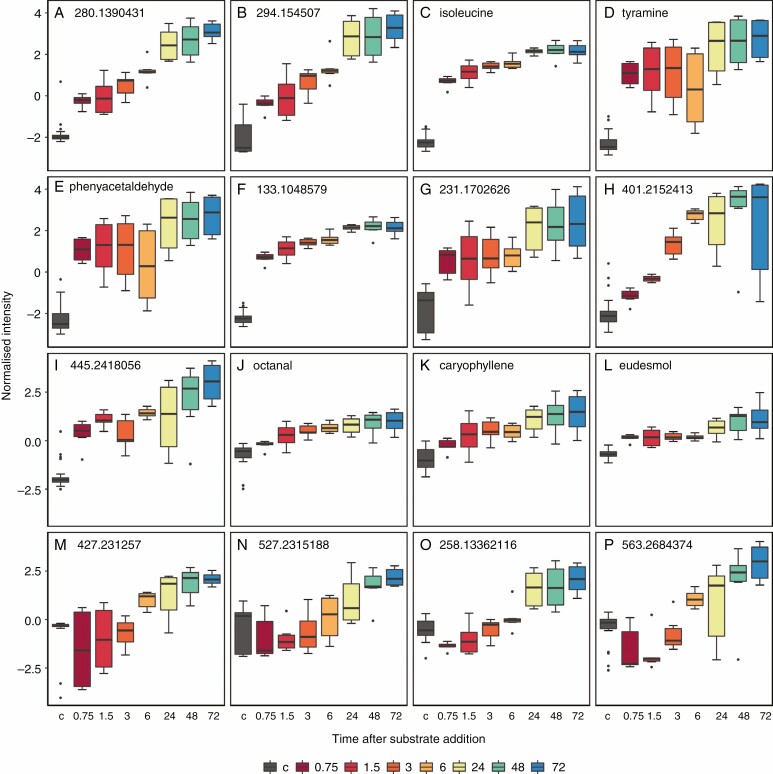
Normalized intensities of filtered metabolites. (A–I) Compounds highlighted as biologically important by all analyses (volcano plot group ≤6 h and ≥24 h and from OPLS-DA of fed groups compared with unfed controls). These compounds identified by OPLS-DA are therefore confirmed by FDR-adjusted *t*-tests likely to be of high biological importance. (C, E, J–L) Compounds previously known to be involved in carnivory and confirmed to be significant by volcano plot in this experiment. (M–P) Compounds identified by three-way OPLS-DA as highly associated with a separation of ≥24 h from unfed controls and plants ≤6 h after feeding. Black (labelled c) (box 1) represents unfed controls (*n* = 32); dark red, red, dark orange and light orange (boxes 2–5) are treatments ≤6 h ; lime green, green and blue (boxes 6–8) are ≥24 h (*n* = 6 per fed treatment harvest time). Note that the *x*-axis is not scaled and therefore distances are not proportional between treatment boxes. Box plots present the median, interquartile range, whiskers are values up to 1.5 times the interquartile range, and values exceeding this are outliers. If a metabolite cannot be annotated, its peak is ascribed.

Compounds identified from fold change and significance (fold change >4 and α < 0.01 from adjusted Welch’s *t*-test) were investigated further to illustrate the general metabolic change in *D. capensis* following prey capture. A heat map with a compound dendrogram shows a clear treatment grouping of controls, followed by the treatments up to 6 h, and then the treatment times >24 h (Fig. 3). There are four clear separations of metabolite patterns. There are compounds that are downregulated 24 h after prey capture, compounds that have a gradual change in concentration over the course of the experiment, and some compounds that appear to have a high increase in concentration initially, followed by a steady increase in concentration for the remainder of the experiment. The results of the volcano plot also confirmed the significance of compounds highlighted in OPLS-DA, a sample of which are included in Fig. 4A–I, M–P.

### Pathway analysis of biologically important metabolites

The compounds involved in carnivory are derived from multiple metabolic pathways (Table 1). Four pathways were found to be significantly involved in the *D. capensis* response to prey addition: flavone and flavonol biosynthesis, phenylalanine metabolism, isoquinoline alkaloid biosynthesis and flavonoid biosynthesis. Sixteen other metabolic pathways were associated with compounds found to significantly change in response to simulated prey capture in this experiment, but these pathways were not statistically significant. They are included here for future work.

**Table 1.** Results of metabolic pathway analysis of *D. capensis* after prey substrate addition. Presented are the number of metabolites in certain metabolic pathways. Statistically significant pathways are in bold type; non-significant pathways are included for reference in future studies. Match status is number of metabolites in this experiment/number of compounds involved in a pathway. Holm *P* is used to control multiple testing. Impact is a combination of centrality and pathway enrichment results calculated as the sum of the importance of each metabolite divided by the sum of all metabolites in each pathway. Statistically significant pathways are indicated in bold.

Pathway	Match status	*P* value	−log(*P*)	Holm *P*	FDR	Impact
**Flavone and flavonol biosynthesis**	**4/9**	**0.0005**	**7·7043**	**0·039226**	**0·039226**	**0·8**
**Phenylalanine metabolism**	**3/8**	**0·0046**	**5·3911**	**0·39189**	**0·19822**	**0·16667**
**Isoquinoline alkaloid biosynthesis**	**2/6**	**0·0284**	**3·5625**	**1**	**0·82268**	**0·5**
**Flavonoid biosynthesis**	**5/43**	**0·0456**	**3·0868**	**1**	**0·99287**	**0·00566**
**Purine metabolism**	5/61	0·1497	1·8992	1	1	0·04869
**Tyrosine metabolism**	2/18	0·2031	1·5942	1	1	0·45455
**Glucosinolate biosynthesis**	4/54	0·2407	1·4244	1	1	0·00952
**Phenylalanine, tyrosine and tryptophan biosynthesis**	2/21	0·2558	1·3635	1	1	0
**Indole alkaloid biosynthesis**	1/7	0·2845	1·2571	1	1	0
**Valine, leucine and isoleucine biosynthesis**	2/26	0·3438	1·0677	1	1	0·01865
**Aminoacyl-tRNA biosynthesis**	4/67	0·3809	0·96519	1	1	0
**Nicotinate and nicotinamide metabolism**	1/12	0·4373	0·82719	1	1	0
**Valine, leucine and isoleucine degradation**	2/34	0·4768	0·74074	1	1	0
**Zeatin biosynthesis**	1/19	0·5987	0·51308	1	1	0
**Ubiquinone and other terpenoid-quinone biosynthesis**	1/23	0·6694	0·40135	1	1	0
**α-Linolenic acid metabolism**	1/23	0·6694	0·40135	1	1	0
**Diterpenoid biosynthesis**	1/26	0·7143	0·33646	1	1	0·01368
**Tryptophan metabolism**	1/27	0·7279	0·31762	1	1	0·17059
**Porphyrin and chlorophyll metabolism**	1/29	0·7532	0·28347	1	1	0·00806
**Glycine, serine and threonine metabolism**	1/30	0·7649	0·26797	1	1	0

### Convergence of compounds for plant carnivory

Thirty-four secondary plant metabolites already known to function in plant carnivory were also present in *D. capensis* in this experiment from putative annotation. Of these, only nonanal (pelargonaldehyde) did not have significant fold changes in concentration after prey substrate addition when compared with the results of the multiple ANOVA. From the volcano plot highlighting high statistical significance and fold changes, five compounds were identified that are also important in carnivory for other carnivorous plants (Fig. 4: C, E, J, K and L). Of the 34 compounds, 21 compounds have been found in *D. capensis* that are also produced and linked to carnivory in plants that do not share a common carnivorous ancestor (light grey shading in Table 2). Eleven compounds have been identified for the first time in Nepenthales (a carnivorous clade of five genera with multiple trap types) that are also present in an unrelated carnivorous lineage (dark grey shadingrows in Table 2).

**Table 2.** Secondary metabolites annotated in this experiment that have been found to have a role in carnivory in a previous study, highlighting occurrence of this compound in other carnivorous plants and whether this experiment shows agreement with previously proposed function. Light-grey bars are metabolites newly found present for *Drosera*. Dark-grey bars are newly found in Nepenthales. White bars are compounds found previously for *D. capensis.* Ticks indicate that findings of this experiment are congruent with the previously proposed function. Crosses do not agree with previously proposed function and brackets indicate partial agreement or disagreement and these compounds require further investigation. ns, no significant fold change across any treatment; arrows indicate concentration increase or decrease by the end of the experiment. No. lineages, number of lineages in which the metabolite has been found to have a carnivorous function. Compounds reported from Hotti *et al.* (2017) and Kreuzwieser *et al.* (2014) have 70% confidence minimum. The proposed function of all metabolites are either known (i.e. directly tested) or highly likely (but not directly or individually tested). Note that some compound isomers are identified, but in some studies this detail is not included. Volatile organic compound (VOC) is ascribed in Proposed function column if detail on the compound’s scent is not available

Proposed role	Metabolite	Proposed function	Support for suggested function in this study	Plant genus/ species previously studied	Family	Order	Source	No. lineages	Regulation after prey capture in this study
Attraction	-Eudesmol	Floral or fruit scent	✗	*Sarracenia* spp.	Sarraceniaceae	Ericales	Hotti *et al.*, 2017	2	↑
Attraction	2-Phenylethanol (phenylethyl alcohol)	Scent (honey, spice, rose, lilac). Attracts a wide range of taxa (downregulated after prey capture in Venus flytrap)	✗	*Dionaea muscipula*, *Nepenthes rafflesiana*, *Sarracenia flava*, *S. leucophylla*, *S. minor*	Droseraceae, Nepenthaceae, Sarraceniaceae	Nepenthales (Caryophyllales), Ericales	Jürgens *et al.*, 2009; Di Giusto *et al.*, 2010; Kreuzwieser *et al.*, 2014	2	↑
Attraction	2-Phenylethyl acetate (phenethyl acetate)	Scent (fruity, sweet)	(✗)	*Nepenthes rafflesiana*, *Sarracenia flava*, *S. leucophylla*	Nepenthaceae, Sarraceniaceae	Nepenthales (Caryophyllales), Ericales	Jürgens *et al.*, 2009; Di Giusto *et al.*, 2010	2	↑
Attraction	5-Hydroxy-methylfurfural	Scent (unknown specific attraction)	✓	*Sarracenia flava*, *S. leucophylla*, *S. minor*	Sarraceniaceae	Ericales	Jürgens *et al.*, 2009	2	↓
Attraction	6-Methyl-5-hepten-2-one	Scent (unknown specific attraction	(✗)	*Dionaea muscipula*, *Drosera binata*, *Nepenthes rafflesiana*, *Sarracenia flava*, *S. leucophylla*, *Sarracenia minor*, *S. purpurea*	Droseraceae, *Nepenthaceae*, Sarraceniaceae	Nepenthales (Caryophyllales), Ericales	Jürgens *et al.*, 2009; Di Giusto *et al.*, 2010; Kreuzwieser *et al.*, 2014	2	↑
Capture, retention and digestion	Abscisic acid	Trap closure sensitivity in Venus flytrap	✓	*Dionaea muscipula*	Droseraceae	Nepenthales (Caryophyllales)	Escalante-Pérez *et al.*, 2011	1	↑
Attraction or capture	Apigenin (and derivatives acacetin and 7,4′-dimethyl ether)	Not tested (present in sticky resin)	(✓)	*Roridula gorgonias*	Roridulaceae	Ericales	Wollenweber, 2007	2	↓
Attraction	Benzaldehyde	Scent (almond, burnt sugar) attracts Lepidoptera, Pieridae). Emitted from *Dionaea muscipula* trap but not individually confirmed to attract prey	✗	*Dionaea muscipula*, *Nepenthes rafflesiana*, *Sarracenia flava*, *S. leucophylla*	Droseraceae, Nepenthaceae, Sarraceniaceae	Nepenthales (Caryophyllales), Ericales	Jürgens *et al.*, 2009; Di Giusto *et al.*, 2010; Kreuzwieser *et al.*, 2014	2	↑
Attraction	Benzyl acetate	Scent (floral; attracts Lepidoptera, Noctuidae)	✗	*Nepenthes rafflesiana*, *Sarracenia flava*, *S. leucophylla*	Nepenthaceae, Sarraceniaceae	Nepenthales (Caryophyllales), Ericales	Jürgens *et al.*, 2009; Di Giusto *et al.*, 2010	2	↑
Attraction	Benzyl alcohol	Sweet, flower scent (emitted from Venus flytrap trap but not individually confirmed to attract prey)	(✗)	*Dionaea muscipula*, *Nepenthes rafflesiana*, *Sarracenia flava*, *S. leucophylla*	Droseraceae, Nepenthaceae, Sarraceniaceae	Nepenthales (Caryophyllales), Ericales	Jürgens *et al.*, 2009; Di Giusto *et al.*, 2010; Kreuzwieser *et al.*, 2014; Hotti *et al.*, 2017	2	↑
Attraction	Caryophyllene oxide	Floral or fruit scent (downregulated after prey capture in Venus flytrap)	✗	*Dionaea muscipula*, *Nepenthes rafflesiana*	Droseraceae, Nepenthaceae	Nepenthales (Caryophyllales)	Di Giusto *et al.*, 2010; Kreuzwieser *et al.*, 2014	1	↑
Capture	Coniine	Insect-paralysing agent	✓	*Darlingtonia californica*, *Heliamphora* spp., *Sarracenia* spp.	Sarraceniaceae	Ericales	Hotti *et al.*, 2017	2	↑
Attraction	Decanal	Floral or fruit scent (emitted from Venus flytrap trap but not individually confirmed to attract prey)	✗	*Dionaea muscipula*, *Nepenthes rafflesiana*	Droseraceae, Nepenthaceae	Nepenthales (Caryophyllales)	Di Giusto *et al.*, 2010; Kreuzwieser *et al.*, 2014	1	↑
Digestion	Gallic acid	Anti-fungal	✓	*Dionaea muscipula*, *Drosera capensis*, *Nepenthes anamensis*	Droseraceae, Nepenthaceae	Nepenthales (Caryophyllales)	Kováčik *et al*., 2012a, b	1	↓
Capture/digestion/retention	Jasmonates (jasmonic acid, 12-oxo-phytodienoic acidic and isoleucine conjugate of jasmonic acid)	Trap seal and secretion of enzymes in *Dionaea muscipula.* Leaf bending in *Drosera* spp. after prey capture. Digestive enzyme secretion in *Drosera* spp., *Dionaea muscipula* and *Nepenthes alata*	✓	*Dionaea muscipula*, *Drosera capensis*, *Nepenthes alata*	Droseraceae, Nepenthaceae	Nepenthales (Caryophyllales)	Escalante-Pérez *et al.*, 2011; Nakamura *et al.*, 2013; Libiaková *et al.*, 2014; Mithöfer *et al.*, 2014; Yilamujiang *et al.*, 2016; Krausko *et al.*, 2017; Pavlovič *et al.*, 2017; Pavlovič and Mithöfer 2019	1	↑
Attraction	Isomenthol	VOC (emitted from trap but not individually confirmed to attract prey)	✗	*Dionaea muscipula*	Droseraceae	Nepenthales (Caryophyllales)	Kreuzwieser *et al.*, 2014	1	↑
Digestion	Isorhamnetin	Not specifically tested	✗	*Drosera adelae*, *D. aliciae*, *D. capensis*, *D. cuneifolia*, *D. ramentacea*	Droseraceae	Nepenthales (Caryophyllales)	Marczak *et al.*, 2005	1	↓
Attraction or capture	Kaempferol	Not tested (present in sticky resin)	(✗)	*Roridula dentata*	Roridulaceae	Ericales	Wollenweber, 2007	2	↓
Attraction	Linalool	Floral, lavender scent. Attracts Lepidoptera, Noctuidae, Hymenoptera	(✗)	*Nepenthes rafflesiana*, *Sarracenia minor*	Nepenthaceae, Sarraceniaceae	Nepenthales (Caryophyllales), Ericales	Jürgens *et al.*, 2009; Di Giusto *et al.*, 2010	2	↑
Attraction or capture	Luteolin (and derivatives velutin and apometzgerin)	Not tested (present in sticky resin)	(✗)	*Roridula gorgonias*	Roridulaceae	Ericales	Wollenweber, 2007	2	↓
Attraction	Methyl salicylate	Scent (peppermint, attracts Lepidoptera, Noctuidae, usually acts as a stress response)	✓	*Dionaea muscipula*, *Nepenthes rafflesiana*, *Sarracenia flava*, *S. leucophylla*	Droseraceae, Nepenthaceae, Sarraceniaceae	Nepenthales (Caryophyllales), Ericales	Jürgens *et al.*, 2009; Di Giusto *et al.*, 2010	2	↓
Digestion	Myricetin	Antibacterial	(✗)	*Dionaea muscipula*, *Drosera adelae*, *D. aliciae*, *D. capensis*, *D. cuneifolia*, *D. ramentacea*	Droseraceae	Nepenthales (Caryophyllales)	Marczak *et al.*, 2005; Krolicka *et al.*, 2008	1	↓
Attraction	Nonanal	Floral scent	ns	*Dionaea muscipula*, *Nepenthes rafflesiana*, *Sarracenia* spp.	Droseraceae, Nepenthaceae, Sarraceniaceae	Nepenthales (Caryophyllales), Ericales	Di Giusto *et al.*, 2010; Kreuzwieser *et al.*, 2014; Hotti *et al.*, 2017	2	ns
Attraction	Octanal	VOC (emitted from trap but not individually confirmed to attract prey)	(✗)	*Dionaea muscipula*	Droseraceae	Nepenthales (Caryophyllales)	Kreuzwieser *et al.*, 2014	1	↑
Attraction	*p*-Cymene	Floral or fruit scent (downregulated after prey capture in Venus flytrap)	✗	*Dionaea muscipula*, *Sarracenia* spp.	Droseraceae, Sarraceniaceae	Nepenthales (Caryophyllales), Ericales	Kreuzwieser *et al.*, 2014; Hotti *et al.*, 2017	2	↑
Attraction	Phenylacetaldehyde	Scent	✗	*Heliamphora* spp.	Sarraceniaceae	Ericales	Jaffé *et al.*, 1995	2	↑
Capture and/or digestion	Plumbagin (or isomer 2-methyljuglone)	Toxic or anaesthetic to prey in *Nepenthes khasiana*. Antibacterial, insecticide	✓	*Aldrovanda vesiculosa*, *Dionaea muscipula*, *Drosera adelae*, *D. aliciae*, *D. auriculata*, *D. binata*, *D. capensis*, *D. cistiflora*, *D. dichotomata*, *D. indica*, *D. longifolia*, *D. lunata*, *D. ramentacea*, *D. whitakeri*, *Drosophyllum lusitanicum*, *Nepenthes gracilis*, *N. khasiana*, *N rafflesiana*, *Triphyophyllum peltatum*	Droseraceae, Drosophyllaceae, Nepenthaceae, Dioncophyllaceae	Nepenthales (Caryophyllales)	Zenk *et al.*, 1969; Cannon *et al.*, 1980; Culham and Gornall, 1994; Bringmann *et al.*, 2000; Rischer *et al.*, 2002; Aung *et al.*, 2002; Marczak *et al.*, 2005; Gonçalves *et al.*, 2008; Krolicka *et al.*, 2008; Raj *et al.*, 2011; Buch *et al.*, 2013	1	↓
Attraction or capture	Quercetin	Not tested (present in sticky resin)	(✓)	*Roridula dentata*	Roridulaceae	Ericales	Wollenweber, 2007	2	↓
Attraction	*Trans*-jasmone	Floral or fruit scent	(✗)	*Sarracenia* spp.	Sarraceniaceae	Ericales	Hotti *et al.*, 2017	2	↑
Attraction or capture	Tricetin (derivative corymbosin)	Not tested (present in sticky resin)	(✓)	*Roridula gorgonias*	Roridulaceae	Ericales	Wollenweber, 2007	2	↓
Attraction	Xylene (*o*-, *m*-, *p*-)	Found on the spoon of the pitcher, assumed to be for attraction	✗	*Heliamphora heterodoxa*, *H. tatei*	Sarraceniaceae	Ericales	Jaffé *et al.*, 1995	2	↑

## DISCUSSION

We have profiled, for the first time, the whole-leaf metabolome response to the addition of animal substrate to a carnivorous plant trap. Though some metabolites have been previously shown to be involved in plant carnivory (Hatcher *et al.*, 2020), we show that there is a substantial metabolic response, which involves a large proportion of known biochemical systems as well as at least 164 unidentified secondary metabolites. The largest response (fold changes and number of metabolites) was of compounds previously associated with defence processes, and compounds that are associated with the attraction of pollinators in other plant systems. This finding is important because it indicates that carnivory, at the metabolic level, is evolved from at least two distinct plant functions: defence and attraction of organisms. Many of the secondary metabolites that demonstrated a statistically significant response had fold increases >30 times the concentration in unfed plants. This demonstrates that there is a clear and substantial metabolic response to prey capture.

Secondary plant metabolites are hypothesized to play an important role in plant diversification and evolution into novel environments (Theis and Lerdau, 2003; Wink, 2003; Lewinsohn and Gijzen, 2009). If secondary metabolites are a conduit for plant evolutionary adaptation, co-occurring metabolites are expected to be present among unrelated taxa that are evolved to occupy similar habitats or possess similar syndromes (Fang *et al.*, 2018). The extent to which convergent evolution of secondary metabolites is true for carnivorous plants is unclear (Ellison and Gotelli, 2009). We identified putatively ~34 compounds present in *D. capensis* that are hypothesized to be involved in carnivory in other carnivorous plants. Eleven of these compounds are new to the Nepenthales lineage but have been previously identified in other carnivorous lineages (Table 2). Two-thirds of these 34 compounds are found across independent lineages of carnivory. It is likely that the same compounds are used to some extent for the purpose of acquiring nutrients from prey, even in different carnivorous plant lineages i.e. phenylacetaldehyde, nonanal and quercetin. These shared metabolic traits, and the clear potential for them to play a role in carnivory, provide some support to the hypothesis that secondary plant metabolites are important for evolutionary adaptation to specific environments (Theis and Lerdau, 2003; Wink, 2003). This remains speculative until confirmation with robust tests of convergent metabolic evolution for plant carnivory. Some shared metabolites perform other functions and are shared with non-carnivorous plants. For example, compounds found only to increase following prey digestion and assimilation may be a consequence of increased nutrient status, rather than holding a role in the carnivorous habit specifically. Additionally, the compounds we identify in this study are annotated from metabolite databases, and therefore require specific targeted analysis to comprehensively confirm their presence, concentration and function.

*Drosera capensis* has co-opted or exapted the use of existing secondary plant metabolites known to function in non-carnivorous plant processes for responding to prey capture (Nakamura *et al.*, 2013; Pavlovič and Saganová, 2015). In the present study, some of these compounds increased to considerably higher concentrations and/or were sustained for much longer in response to prey capture than is typical of the known response in non-carnivorous plant functions. Defence-related jasmonates such as isoleucine increased by 3000 % within 24 h of simulated prey capture and were sustained above this level for the full 72-h experiment. Wounding has been shown to instigate a transient, comparatively low change in concentration of jasmonates, and the response of this compound to herbivory peaks similarly to the response to prey capture after 1.5 h but returns to the original concentration by 3 h post-herbivory (Mithöfer *et al.*, 2014). Furthermore, we measured 36-fold increases in the floral scent and flavour phenylacetaldehyde in response to prey capture. Metabolites can have multiple functions within the plant based on biological thresholds. Here, *D. capensis* appears to have metabolic thresholds for this compound at much higher levels in response to prey capture than has been demonstrated in other plants for different functions. For example, in tomato (*Solanum lycopersicum*), overexpression of genes controlling phenylacetaldehyde increased concentration by up to ten times (Schwab *et al.*, 2008). It is therefore clear that in *D. capensis* prey capture instigates a highly complex biochemical response utilizing many compounds across multiple metabolic pathways, differentiating from non-carnivorous functions for the same compounds through either larger fold changes or by sustaining high levels for longer.

The increased concentration of metabolites following prey capture is possibly a result of a compound’s function in carnivory, but alternatively may be the result of increased nutrient status following prey digestion. *Nepenthes insignis* directly incorporated C2 units, from a solution of sodium acetate and alanine added to the prey-capturing pitchers, for plumbagin synthesis (Rischer *et al.*, 2002). Such metabolic responses have also been identified in non-carnivorous plants, for example *Plumbago indica* (Jaisi and Panichayupakaranant, 2017). That some metabolites, in this study, had an initial statistically significant response within hours suggests a metabolic response to prey capture rather than because of altered nutrient status. Additionally, some metabolites showed a dynamic response in concentration to prey capture i.e. an initial decrease, followed by a gradual increase in concentration over time (Fig. 4P). These results suggest that many metabolites identified here are a response to prey capture. We cannot, however, rule out the possibility that some metabolite concentration changes may simply be a result of a change in plant nutrient status following prey digestion and assimilation. Thus, further investigation into the function of specific metabolites identified in this study is necessary.

The function of compounds that decrease in concentration may have multiple biological explanations and should be interpreted with caution. Many metabolites in plants are stored as an inactive compound until some form of stimulus instigates the activation of these compounds at specific times, often through glycosylation (Gachon *et al.*, 2005). Important compounds can, as a result, rapidly become active rather than the plant having to synthesize the whole compound on demand. The presentation of these processes would be of one metabolite increasing in concentration whilst another decreases. While it remains reasonable to state that compounds up- and downregulated following prey capture are likely to be involved in some aspect of carnivory, their specific function, particularly for fold decreases, cannot be explicitly determined. It can be stated, however, that due to the high number of compounds involved that change in intensity following prey capture (~2383 features), there is a reliance on secondary plant metabolites to facilitate plant carnivory.

Our results provide a clear focus for future studies, in particular the role of volatile organic compounds (VOCs). Kreuzwieser *et al.* (2014) hypothesize that VOCs are downregulated following prey capture and speculate that this is because if the compound functions as an attractant to prey it is not necessary to produce it following prey capture. Reduced synthesis of attractants following prey capture is a logical response because a decrease in the production of these compounds minimizes the net cost of production, which may reduce the cost of carnivory (Ellison and Gotelli, 2009; Grace and Hudson, 2016). Evidence from our study is, however, equivocal to Kreuzwieser *et al.* (2014), as only a subset of identified VOCs decreases following insect substrate addition (e.g. isorhamnetin, tricetin, gallic acid and kaempferol; Table 2). These differences may be explained by sampling approaches; Kreuzwieser *et al.* (2014) measured volatiles emitted from the leaf, as opposed to, in our study, directly measuring the leaf. Further work is necessary to determine the role of some VOCs in carnivory and may require GC-MS to identify highly volatile VOCs.

The majority (142 of ~170) of secondary plant metabolites involved in carnivory are classified as VOCs and are assumed to be attractants for prey (see review by Hatcher *et al.*, 2020). The assumption that VOCs attract prey may be misleading. Rather than a description of biological relevance, the classification of VOCs is a chemical grouping and these compounds usually have very low volatility under ambient conditions (under European classification VOCs have boiling points <250 °C; US EPA, 2019). In the present experiment, 24 of the 34 compounds also found in other carnivorous plants have previously been suggested to function in attraction (Table 2) (Jürgens *et al.*, 2009; Kreuzwieser *et al.*, 2014; Hotti *et al.*, 2017). If compounds are involved in attraction, it is expected that these compounds will decrease in concentration after prey capture, or at the very least not change in concentration, as found in a study on prey-induced changes to phenolic metabolites in *D. capensis* (Kováčik *et al.*, 2012*b*). Of these 24 compounds, however, 18 increase in concentration after prey capture, in some cases by >30 times the concentration in unfed plants (Table 2). This is contradictory to expected responses if the compound is solely involved in the attraction of prey. Such findings may be in contrast to the findings of Kováčik *et al*. (2012b) due to differences in prey addition method (powdered flies or whole ants) and temporal sampling. These differences may mean certain transient changes in metabolites are not recorded or responses instigated in this experiment do not occur with different prey addition techniques. Involvement of these 24 compounds in carnivory is not disputed here. We propose, however, that these compounds provide an alternative function, at least for *D. capensis* but probably also for other carnivorous plants, that aids in capture, retention, digestion or assimilation. Volatile organic compounds require further investigation to establish their biological function, rather than relying solely on chemical classification (Table 2).

We conclude that secondary plant metabolites have a substantial role in the multifaceted plant adaptation of carnivory in *D. capensis*. Response to prey capture involves a whole-metabolome response. Not only does it appear that there is a strong biochemical basis in response to prey capture, but there also appears to be a large diversity of compounds – larger than previously considered – that are important for carnivory in plants. Secondary metabolites may be much more important for plant carnivory than previously thought. In addition, we provide evidence that there is convergence in the secondary metabolites involved in carnivory across independently evolved lineages of carnivorous plant. This work supports the hypothesis of Wagner (2017) that secondary metabolites may therefore be important drivers of plant evolution and diversification into new environments. Future work should focus on characterisation of the metabolomic response across species of carnivorous plant, and identification of biologically relevant metabolites and their functions.

## SUPPLEMENTARY DATA

Supplementary data are available online at https://academic.oup.com/aob and consist of the following. Methods Detail: extractions, UHPLC-MS analysis and data processing. Figure S1: total ion current (TIC) chromatogram for a blank and a QC run. Figures S2–S9: OPLS-DA S-plot and score plots. Table S1: cross-comparison of univariate and OPLS-DA analysis. Table S2: FDR-adjusted ANOVA of fed plants compared with unfed controls.

mcab065_suppl_Supplementary_Figures

mcab065_suppl_Supplementary_Materials

mcab065_suppl_Supplementary_Table_S1

mcab065_suppl_Supplementary_Table_S2
