# Supplementary material for: Metabolomic analysis reveals reliance on secondary plant metabolites to facilitate carnivory in the Cape sundew, Drosera capensis
Source: Ann Bot. 2021 Jun 2;128(3):301–14. doi: 10.1093/aob/mcab065 (PMC8389465; doi:10.1093/aob/mcab065)
Supplement: mcab065_suppl_Supplementary_Figures [file mcab065_suppl_supplementary_figures.docx]

***Supplementary figures:***

 Figure S1: Total ion current (TIC) chromatogram for a blank and a QC run.

Figure S2: Orthogonal partial least squares-discriminant analysis model 2-dimensional score plot for unfed plants (black circles) and plants up to 6 hrs after feeding (green triangles). Hotelling’s t^2^ as a quality check to see all the data are within normal boundaries. Ellipsoid is 95% confidence interval for the data.

Figure S3: S-plot for modelled variables using an orthogonal partial least squares-discriminant analysis model with unit variance scaling for unfed versus ‘≤ 6 hrs’ after feeding samples. Important compounds are highlighted with red ellipses.

Figure S4: Orthogonal partial least squares-discriminant analysis model 2-dimensional score plot for unfed plants (black circles) and plants ‘≥ 24 hrs’ after feeding (blue squares). Hotelling’s t^2^ as a quality check to see all the data are within normal boundaries. Ellipsoid is 95% confidence interval for the data.

Figure S5: S-plot for modelled variables using an orthogonal partial least squares-discriminant analysis model with unit variance scaling for unfed versus ‘≥ 24 hrs’ after feeding samples. Important compounds are highlighted with red ellipses.

Figure S6: Orthogonal partial least squares-discriminant analysis model 2-dimensional score plot for plants ‘≤ 6 hrs’ after feeding (green triangles) and plants ‘≥ 24 hrs’ after feeding (blue squares). Hotelling’s t^2^ as a quality check to see all the data are within normal boundaries. Ellipsoid is 95% confidence interval for the data.

Figure S7: S-plot for modelled variables using an orthogonal partial least squares-discriminant analysis model with unit variance scaling for plants ‘≤ 6 hrs’ after feeding versus plants ‘≥ 24 hrs’ after feeding. Important compounds are highlighted with red ellipses.

Figure S8: Orthogonal partial least squares-discriminant analysis model 2-dimensional score plot for unfed plants (black circles), plants up to 6 hrs after feeding (green triangles) and plants 24 hrs or later after feeding (blue squares). Hotelling’s t2 as a quality check to see all the data are within normal boundaries. Ellipsoid is 95% confidence interval for the data.

Figure S9: Loading plot of metabolites (X) with relatedness to (Y) plants ‘≥ 24 hrs’ after feeding, ≤6 hrs after feeding and unfed controls (left to right respectively). Red dots are metabolites most associated with plants ‘≥ 24 hrs’ after feeding compared to unfed and ‘≤ 6 hrs’ after feeding.
