# Supplementary material for: Metabolomic analysis reveals reliance on secondary plant metabolites to facilitate carnivory in the Cape sundew, Drosera capensis
Source: Ann Bot. 2021 Jun 2;128(3):301–14. doi: 10.1093/aob/mcab065 (PMC8389465; doi:10.1093/aob/mcab065)
Supplement: mcab065_suppl_Supplementary_Materials [file mcab065_suppl_supplementary_materials.docx]

**Supplementary methods detail for:**

**Metabolomic analysis reveals reliance on secondary plant metabolites to facilitate carnivory in the Cape sundew, *Drosera capensis***

Christopher R. Hatcher^*1,2^, Dr Ulf Sommer^3^, Dr Liam Heaney^2^, Dr Jonathan Millett^2^

1. Loughborough University, Loughborough, England, LE11 3TU
2. Agri-Tech Centre, Pershore College, Part of WCG, England, WR10 3JP
3. Biocrates Life Sciences AG, 6020, Innsbruck, Austria

* Corresponding author:

Email: chris.hatcher@btinternet.com

Telephone: 07791358828

Running title: Metabolomic analysis reveals reliance on metabolites to facilitate plant carnivory

Supplementary material

***Supplementary detail of methods***

Monophasic extractions used CK-14 Precellys homogeniser tubes and acetonitrile:methanol:water 2:2:1 as the final solvent. Differences in weight of tissue was accounted for by using a fixed amount of the original homogenate, set by the smallest sample (14.9 mg). After centrifugation, equal volumes of the supernatant were transferred into new glass vials and dried in a SpeedVac before storage at −80°C. LC-MS samples were reconstituted in 50 µl, 20% aqueous methanol each, and 10 µl each were pooled into one Quality Control (QC) sample per sample type, 16 QC samples in total. All solvents were of LC-MS grade.

### UHPLC-MS analysis (positive ion mode)

After centrifugation, (15000 rpm for 10 min at 4°C, Biofuge), 20 µl per sample were pipetted into a 96-well plate, starting with a blank, QC samples, samples with intermittent further QC samples, and ending with a blank after two QC samples. The samples were run in controlled randomised order, with QC samples equidistant between them. They were analysed by Ultra High Performance Liquid Chromatography-Mass Spectrometry (UHPLC-MS) on a Thermo Scientific Q Exactive mass spectrometer interfaced with a Thermo Dionex Ultimate 3000 RS system, equipped with a Thermo Hypersil Gold column (100 x 2.1 mm, 1.9 µm particles, C18 material). Solvents: (A) 0.1% formic acid in water and (B) 0.1% formic acid in methanol. Method: 14 min LC run with a constant flow of 400 µl / min starting at 100% A and held for 1 min, reducing linearly to 0% A over 7 min and held at 0% A for 3 min before returning to 100% A over 1 min and equilibrating for a further 3 min before the next injection, , with 18 µl injections per sample and MS start at 0.1 min, with the flow up to 0.45 min directed towards waste. Quality Control sample 02 contains data-dependent tandem mass spectrometry (MS/MS) data and is used with the retention time index to annotate metabolites and another QC sample was run at 140k resolution. Data were collected in positive ion and profile mode, *m/z* 100-1000, at 70k resolution (See Supplementary Fig. 1 for total ion chromatogram for a blank and QC run).

### Data processing

Data processing and quality control methods followed guidelines outlined by Guida *et al.* (2016) and Kirwan *et al.* (2014) using NBAF-B in house scripts in MatLab (v8.1; The MathWorks, Natick, MA, USA), the SIMStitch pipeline. Briefly, Thermo .raw data files in profile mode were converted into mzML files in centroid mode using MSConvert (Proteowizard 3.0.7665). An R (3.2.0) based XCMS / CAMERA script was used for alignment and resulted in an intensity matrix in a csv file (9309 features). The matrix was imported into MatLab and inserted into a SIMStitch pipeline. During replicate filtering, peaks were retained if they were found in two out of three technical replicates. A blank filter of > 2× sample over blank signal was applied and a sample filter of peak presence of at least 75% of all samples. Samples were filtered with a 2-ppm mass error and a 75% filter. Data were normalised using probabilistic quotient normalisation (PQN) and missing values being filled using the k-nearest neighbours (KNN) algorithm (k=5). This matrix was used for univariate statistics including fold-changes. Generalised logarithm (g-log) transformation was optimised on QC samples only and then applied to all samples. This matrix was used for multivariate statistics (over Pareto scaling as there is less bias towards more intense signals). Normalisation and g-log transformation are applied to make the samples more comparable and to reduce heteroscedasticity.

The XCMS matrix and therefore the dataset object for the samples contained 5423 analytical features, the blank filtered dataset object had 3259 peaks, and the sample filtered dataset object had 3257 peaks. The median relative standard deviation (RSD) for the QC samples in the normalised dataset was 7.48 % (without QC01-QC04). This confirms the high technical quality of this dataset (for biomarker studies, the FDA guidelines specify an RSD of < 20% as an acceptable level of precision and presenting as a median RSD is the best practice, (Parsons *et al.* 2009; Kirwan *et al.* 2014).
