# Supplementary material for: Metabolomic analysis reveals reliance on secondary plant metabolites to facilitate carnivory in the Cape sundew, Drosera capensis
Source: Ann Bot. 2021 Jun 2;128(3):301–14. doi: 10.1093/aob/mcab065 (PMC8389465; doi:10.1093/aob/mcab065)
Supplement: mcab065_suppl_Supplementary_Table_S1 [file mcab065_suppl_supplementary_table_s1.docx]

Supplementary Table 1: Cross comparison of univariate and OPLS-DA analysis. T-tests confirm grouping from OPLS-DA. Only annotated compounds included in this table. This table was used for metabolic pathway analysis.

| Putative annotation | 24 to C  t-test | 6 to C  t-test | OPLS-DA  6 TO C | OPLS-DA 24 TO C | OPLS-DA  24 to 6 | OPLS-DA  24 to 6 & C | Regulation |
| --- | --- | --- | --- | --- | --- | --- | --- |
| Isoleucine | ✓ | ✓ | ✓ | ✓ |  |  | ↑ |
| Phenylacetaldehyde | ✓ | ✓ | ✓ | ✓ |  |  | ↑ |
| Tyramine | ✓ | ✓ | ✓ | ✓ |  |  | ↑ |
| 1-(3-aminopropyl)-pyrrolinium | ✓ | ✓ | ✓ |  |  |  | ↑ |
| 8-Oxodeoxycoformycin | ✓ |  | ✓ | ✓ |  |  | ↓ |
| Carnitine isomer | ✓ | ✓ | ✓ |  |  |  | ↑ |
| Choline sulphate | ✓ | ✓ | ✓ |  |  |  | ↑ |
| D-Methionine | ✓ | ✓ | ✓ |  |  |  | ↑ |
| Hypoxanthine | ✓ | ✓ | ✓ |  |  |  | ↑ |
| Idanpramine | ✓ | ✓ | ✓ |  |  |  | ↑ |
| Methionine oxide | ✓ | ✓ | ✓ |  |  |  | ↑ |
| Napthylamine isomer | ✓ | ✓ | ✓ |  |  |  | ↑ |
| SR 12813 | ✓ |  |  |  | ✓ | ✓ | ↑ |
| Tryptophan | ✓ | ✓ | ✓ |  |  |  | ↑ |
| Xanthine | ✓ | ✓ | ✓ |  |  |  | ↑ |
| 1-(3-Aminopropyl)-4-aminobutanal | ✓ |  |  |  | ✓ |  | ↑ |
| 11-Aminoundecanoic acid |  | ✓ | ✓ |  |  |  | ↓ |
| Butabarbital | ✓ | ✓ |  |  |  |  | ↑ |
| Dethiobiotin | ✓ | ✓ |  |  |  |  | ↑ |
| Glycyl-leucine | ✓ | ✓ |  |  |  |  | ↑ |
| Guanosine isomer | ✓ | ✓ |  |  |  |  | ↑ |
| Ipratropium | ✓ | ✓ |  |  |  |  | ↑ |
| Leucyl-leucine | ✓ | ✓ |  |  |  |  | ↑ |
| Linmarin | ✓ | ✓ |  |  |  |  | ↑ |
| Rhizocticin isomer | ✓ |  |  | ✓ |  |  | ↓ |
| Tryptophan isomer | ✓ | ✓ |  |  |  |  | ↑ |
| Tyrosine | ✓ | ✓ |  |  |  |  | ↑ |
| (2S,3S)-2-Hydroxytridecane-1,2,3-tricarboxylate | ✓ |  |  |  |  |  | ↑ |
| 11-Aminoundecanoic acid | ✓ |  |  |  |  |  | ↑ |
| 2-Phenylacetamide | ✓ |  |  |  |  |  | ↑ |
| 3-Oxododecanoic acid | ✓ |  |  |  |  |  | ↑ |
| 5-Methylthioadenosine | ✓ |  |  |  |  |  | ↑ |
| Acetylagmatine | ✓ |  |  |  |  |  | ↑ |
| Adenine | ✓ |  |  |  |  |  | ↑ |
| Benzoylagmatine | ✓ |  |  |  |  |  | ↑ |
| Buchananine | ✓ |  |  |  |  |  | ↑ |
| Caryophyllene oxide (epoxide) | ✓ |  |  |  |  |  | ↑ |
| cis-2-Carboxycyclohexyl-acetic acid | ✓ |  |  |  |  |  | ↑ |
| CPX | ✓ |  |  |  |  |  | ↑ |
| Crotono-betaine | ✓ |  |  |  |  |  | ↑ |
| Cycloate | ✓ |  |  |  |  |  | ↑ |
| D-lyposine | ✓ |  |  |  |  |  | ↑ |
| Danorubicin |  |  | ✓ |  |  |  | ↓ |
| Dibutyl adipate | ✓ |  |  |  |  |  | ↑ |
| Eudesmol / nerolidol | ✓ |  |  |  |  |  | ↑ |
| Fagomine | ✓ |  |  |  |  |  | ↑ |
| Fortimicin isomer | ✓ |  |  |  |  |  | ↑ |
| Gibberellin A41 | ✓ |  |  |  |  |  | ↑ |
| Guanine | ✓ |  |  |  |  |  | ↑ |
| HC-toxin | ✓ |  |  |  |  |  | ↑ |
| Hexahomomethionine | ✓ |  |  |  |  |  | ↑ |
| Isoleucine isomer | ✓ |  |  |  |  |  | ↑ |
| Kynurenine | ✓ |  |  |  |  |  | ↑ |
| Leu-Gly-Pro | ✓ |  |  |  |  |  | ↑ |
| Leucine | ✓ |  |  |  |  |  | ↑ |
| Lycodine | ✓ |  |  |  |  |  | ↑ |
| Muramic acid | ✓ |  |  |  |  |  | ↑ |
| N1-Acetylspermine | ✓ |  |  |  |  |  | ↑ |
| Naphthoquinone |  |  | ✓ |  |  |  | ↓ |
| Nicotinate | ✓ |  |  |  |  |  | ↑ |
| Nifuradene | ✓ |  |  |  |  |  | ↓ |
| Octanal | ✓ |  |  |  |  |  | ↑ |
| Octhilinone | ✓ |  |  |  |  |  | ↑ |
| Pantetheine | ✓ |  |  |  |  |  | ↑ |
| Phenazocine | ✓ |  |  |  |  |  | ↑ |
| Phenylethylamine | ✓ |  |  |  |  |  | ↑ |
| Pheophorbide a | ✓ |  |  |  |  |  | ↑ |
| Pirbuterol | ✓ |  |  |  |  |  | ↑ |
| Proacacipetalin | ✓ |  |  |  |  |  | ↑ |
| Quinoline isomer | ✓ |  |  |  |  |  | ↑ |
| Rhododendrin | ✓ |  |  |  |  |  | ↑ |
| Schizonepetoside E | ✓ |  |  |  |  |  | ↑ |
| Spironolactone | ✓ |  |  |  |  |  | ↑ |
| Styrene | ✓ |  |  |  |  |  | ↑ |
| Succinic anhydride | ✓ |  |  |  |  |  | ↑ |
| UCL 1608 | ✓ |  |  |  |  |  | ↑ |
| Xanthopterin-B2 | ✓ |  |  |  |  |  | ↑ |
